# Building Bridges for Collective Wisdom

**DOI:** 10.1200/JGO.2015.000737

**Published:** 2015-09-23

**Authors:** Peter P. Yu, Julie M. Vose

**Affiliations:** Palo Alto Medical Foundation, Palo Alto, CA; University of Nebraska Medical Center, Omaha, NE

Welcome to the inaugural issue of *Journal of Global Oncology* (*JGO*), ASCO's new online-only, open-access journal dedicated to publishing the highest quality information concerning oncology research and care delivery in countries with limited health care resources.

It is estimated that nearly two thirds of all cancer deaths occur in low- and middle-resource countries (LMRCs), and this is forecast to increase to 70% by 2030.^1^ The presidential theme of past ASCO President Sandy Swain was “Building Bridges,” and global health was a major theme of ASCO's 2013 Annual Meeting. ASCO can indeed build bridges that serve to translate the work we do in the United States and other higher resource nations to improve cancer care in other countries. We can also build bridges that improve cancer care for health care disparity populations within developed nations by learning from the experiences of LMRCs that reflect the unique biologic and social attributes of these populations.^2^ This journal is a step toward building those bridges by enabling ASCO members to contribute to the vitally important and dynamic global conversation on health care, including access to care, quality of care, and perhaps most important, the design of systems of health care delivery that affect populations of patients with cancer.

International membership is the fastest growing category for ASCO. In fact, 31% of ASCO members practice outside the United States. Certainly all oncologists, regardless of where in the world they practice, share similar problems and concerns. US physicians have much to learn and gain from the research of our colleagues in other countries, and heretofore there had been a void in the literature of peer-reviewed research and expert commentary on the challenges of LMRCs. While ASCO's flagship *Journal of Clinical Oncology* and its sister *Journal of Oncology Practice* receive many submissions from around the world each year and have published special article series or collections about global oncology research in the past, there presently—or rather, surprisingly—is no journal that focuses solely on this important topic. *JGO* will fill this void. Our hope is that this journal will be the voice oncology professionals with a global connection or interest seek out for the latest practice-changing research and perspectives on cancer care, research, and care delivery issues unique to countries and settings with limited health care resources.

Already there is much good research coming from our international colleagues, but we lack a journal with a dedicated focus on global health. A manuscript with good information for the global oncology community may be rejected because of its limited relevance to the majority of readers of journals with a more general oncology focus. This means that vital information is not being published and getting to our oncology colleagues who need it. *JGO* is eager to serve as a new home for this important work and serve as the channel by which to deliver it to all who are involved in cancer care. Because submissions from authors in LMRCs should be the lifeblood of *JGO*, ASCO will be providing more educational programs for researchers in these countries to support the conduct of meritorious clinical research and the successful submission of manuscripts to peer-reviewed journals.

Professor David Kerr of Oxford University will be at the helm of this new venture and brings a world of prestige and expertise to the position, having published many peer-reviewed articles and founded global oncology initiatives in India and Africa. He also has served on the editorial boards of several oncology journals, including the *Annals of Oncology*, where he served as Editor-in-Chief. Professor Kerr is a Fellow of the Royal College of Physicians, Honorary Fellow of the Royal College of General Practitioners, Fellow of the Academy of Medical Sciences, and Fellow of the European Academy of Cancer Sciences.

As well, we are proud of *JGO*'s associate editors and editorial board members, who bring to the journal multidisciplinary expertise and firsthand clinical practice and research experience from around the world. We are honored and excited to watch this capable team grow ASCO's latest journal. We look forward to seeing *Journal of Global Oncology* discussed among colleagues at our various meetings around the world.

Thank you, reader, for sharing in this first issue. You are here as *JGO* embarks on an exciting new venture. We look to you to send the journal your ideas for content. If *JGO* has not yet published something on a topic you consider current and vital to the internationally based oncologist, please let Dr Kerr know. This journal will look to international experts in the field of oncology to shed light on the challenges of cancer care and research in LMRCs through peer-reviewed original research and editorials. Some of these experts will be your friends. We ask you to encourage them to consider *Journal of Global Oncology* as a home for their important work.

As our population continues to diversify, so too will our oncology workforce and the patients they serve. The creation of *Journal of Global Oncology* is indicative of ASCO's determination to take a lead role in oncology care across the globe by ensuring that oncologists in all settings are well-equipped with contemporary information and practice guidelines, and supports the Society's vision, as we all do, to help improve cancer care and delivery in LMRCs.
